# *Haemophilus influenzae* Protein D antibody suppression in a multi‐component vaccine formulation

**DOI:** 10.1002/2211-5463.13498

**Published:** 2022-10-28

**Authors:** Lea V. Michel, Ravinder Kaur, Michael L. Gleghorn, Melody Holmquist, Karin Pryharski, Janai Perdue, Seth P. Jones, Niaya Jackson, Isabelle Pilo, Anna Kasper, Natalie Labbe, Michael Pichichero

**Affiliations:** ^1^ School of Chemistry and Materials Science Rochester Institute of Technology NY USA; ^2^ Center for Infectious Diseases and Immunology Rochester General Hospital Research Institute NY USA; ^3^ National Technical Institute for the Deaf Rochester Institute of Technology NY USA

**Keywords:** acute otitis media, antibody suppression, antigenic competition, *Haemophilus influenzae*, outer membrane protein 26, Protein D

## Abstract

Nontypeable *Haemophilus influenzae* (*NTHi*) has emerged as a dominant mucosal pathogen causing acute otitis media (AOM) in children, acute sinusitis in children and adults, and acute exacerbations of chronic bronchitis in adults. Consequently, there is an urgent need to develop a vaccine to protect against *NTHi* infection. A multi‐component vaccine will be desirable to avoid emergence of strains expressing modified proteins allowing vaccine escape. Protein D (PD), outer membrane protein (OMP) 26, and Protein 6 (P6) are leading protein vaccine candidates against *NTHi*. In pre‐clinical research using mouse models, we found that recombinantly expressed PD, OMP26, and P6 induce robust antibody responses after vaccination as individual vaccines, but when PD and OMP26 were combined into a single vaccine formulation, PD antibody levels were significantly lower. We postulated that PD and OMP26 physiochemically interacted to mask PD antigenic epitopes resulting in the observed effect on antibody response. However, column chromatography and mass spectrometry analysis did not support our hypothesis. We postulated that the effect might be *in vivo* through the mechanism of protein vaccine immunologic antigenic competition. We found when PD and OMP26 were injected into the same leg or separate legs of mice, so that antigens were immunologically processed at the same or different regional lymph nodes, respectively, antibody levels to PD were significantly lower with same leg vaccination. Different leg vaccination produced PD antibody levels quantitatively similar to vaccination with PD alone. We conclude that mixing PD and OMP26 into a single vaccine formulation requires further formulation studies.

## Graphical abstract

When mice are injected with *NTHi* protein vaccine candidates Protein D or OMP26, there is a robust antibody response to each protein. However, when Protein D and OMP26 are mixed into a single vaccine formulation, mice fail to produce antibody to Protein D. We propose antibody suppression results from a physiochemical interaction or antigenic competition between the two proteins.

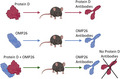

**Abbreviations**
alum: aluminum hydroxide
AOM: acute otitis media
COPD: chronic obstructive pulmonary disease
*E. coli*
: *Escherichia coli*
ELISA: enzyme‐linked immunosorbent assay
*His‐tag*
: Histidine tag
HRP: horseradish peroxidase
i.m.: intramuscular
i.p.: intraperitoneal
MS: mass spectrometry
*NTHi*
: nontypeable *Haemophilus influenzae*
OMP: outer membrane protein
PBS: phosphate‐buffered saline
PCV: pneumococcal conjugate vaccine
SEC: size exclusion chromatography
TBS: tris‐buffered saline
TMB: 3,3′,5,5′‐Tetramethylbenzidine

Nontypeable *Haemophilus influenzae (NTHi*) has become the most common cause of acute otitis media (AOM) in children in the US, persistent AOM, and recurrent AOM [1, 2, 3, 4, 5]. Treatment of AOM in children has an annual cost of over $6 billion in the US [6, 7]. *NTHi* also causes acute sinusitis and conjunctivitis in children and adults and acute exacerbations of chronic obstructive pulmonary disease (COPD) in adults [8, 9, 10, 11]. COPD is the third leading cause of death in the US, affecting at least 24 million people, and US healthcare costs exceed $50 billion each year [12, 13]. These statistics, combined with rising concern over antibiotic‐resistant superbugs, point to the critical need for a vaccine to prevent *NTHi* infections [14, 15]. Many potential *NTHi* vaccine candidates are currently being evaluated by different groups for their protection efficacy in pre‐clinical animal models. The most promising candidates include Proteins D, E, and F, OMP26, P4, P6, PilA, Hap, HMW, and ZnuA [16, 17, 18, 19]. Among these candidates, our group has focused on Protein D (PD), OMP26, and P6 [18, 20, 21, 22, 23, 24], and in this study, we focus on PD and OMP26 as a vaccine mixture.

Protein D is a highly conserved 42‐kDa outer membrane IgD‐binding lipoprotein found in all *H. influenzae* strains [18, 19, 25]. PD exhibits glycerophosphodiesterase activity, causing the release of glycerophosphorylcholine from host epithelial cells, and is therefore thought to be a virulence factor [18, 21]. PD from *NTHi* was included in an investigational vaccine (PCV‐11) as a carrier protein, and results from one study demonstrated that the vaccine reduced AOM caused by *NTHi* by about 35% [26, 27]. Subsequent studies using the current PHiD‐CV vaccine (*i.e*., PCV‐10, not approved in the US), which contains PD conjugated to 8 of the 10 polysaccharides, showed 15–25% vaccine efficacy against *NTHi*‐caused AOM [19, 25, 28]. In addition, animals immunized with PD showed significant protection against *NTHi*‐caused AOM and clearance in middle ears and lungs, corroborating the hypothesis that PD is immunogenic and capable of producing protective antibodies [18].

OMP26 is a 26‐kDa protein that is highly conserved and present in all known *H. influenzae* strains [27]. The function of the protein is currently unknown, but OMP26 shows structural similarities to Skp bacterial proteins, which are molecular chaperones, helping to maintain the solubility and proper fold of outer membrane proteins (OMPs). Rats immunized with OMP26 produced significant titers of IgG, IgA, and IgM to OMP26 in sera and showed significant bacterial lung clearance (compared to sham mice) when challenged with *NTHi* [27]. Pre‐challenge immunization of chinchillas with OMP26 caused rapid clearance of *NTHi* from the nasopharynx and reduced bacterial loads in the middle ear, and OMP26 antibodies have been detected in the sera of children colonized with *NTHi* in their nasopharynx [24, 29]. These studies and others have placed OMP26 as a leading vaccine candidate for *NTHi*.

Formulation of multi‐component protein vaccines is commonly pursued to reduce the risk of vaccine‐induced immune pressure, leading to selection of bacterial escape mutants. For example, surveillance after the pneumococcal polysaccharide PD‐conjugate vaccine (PHiD‐CV) was introduced, that included PD in the vaccine, found that between 5% and 8% of the *H. influenzae* isolates in the nasopharynx or middle ear lacked the *protein D* gene [30, 31]. Having several antigenic components in a vaccine improves strain coverage and reduces risk of emergence of strains escaping vaccine protection. In addition, a multi‐subunit vaccine elicits a *cumulative* immunogenic response to the antigens that enhances overall protection.

However, multi‐component protein vaccine formulations can be complicated by physiochemical interactions or antigenic competition, resulting in masking of key antigenic epitopes and consequent reduced immune responses to one or both ingredient proteins. In the course of our pre‐clinical studies with PD, OMP26, and P6 as vaccine candidates, we found that compositions of PD mixed with OMP26 resulted in reduced PD antibody responses, whereas compositions of PD with P6 did not. Here, we describe experiments evaluating physiochemical interactions and antigen competition mechanisms that might explain the observed reduced immune response to PD when mixed with OMP26.

## Materials and methods

### Recombinant expression and purification of Protein D, OMP26, and P6

PHiD‐CV includes PD in its non‐lipidated form. Therefore, the *protein D* gene with the N‐terminal signal sequence removed was purchased from GenScript and subcloned into a pET21a vector to generate non‐lipidated PD for our experiments. The *omp26* gene was also purchased from GenScript and subcloned into a pET21a vector. His‐tagged versions (N‐terminal 6xHistidine tags) of both genes were also purchased from GenScript. All recombinant versions of PD and OMP26 were expressed in *Escherichia coli* (*E. coli*) BL‐21* cells, induced with IPTG, and purified on Talon Cobalt resin (Clontech) using the proteins' N‐terminal His‐tags. The *NTHi p6* gene in the pET28‐a (Kanamycin resistant) vector was a generous gift from Dr. John Orban (University of Maryland Biotechnology Institute). P6 protein in non‐lipidated form with the 19 residue N‐terminal signal sequence removed and including a His‐tag at the N terminus was also expressed in *E. coli* and purified on Talon resin.

#### Cobalt affinity purification

*Escherichia coli* cells expressing either the His‐tagged proteins or the non‐tagged proteins were lysed using sonication, and cell lysates were incubated (for 1 h at room temperature or overnight at 4 °C) with the Talon Cobalt beads individually or in combination. The Talon resin was washed with Equilibration/Wash buffer, as recommended by the manufacturer. The proteins were eluted in 150 mm imidazole (Elution buffer, recommended by the manufacturer). The eluted fractions that contained protein were combined, boiled in 2× SDS/PAGE sample buffer (2× recipe: 0.12 m Tris/HCl pH 6.8, 4% SDS, 20% glycerol, 0.01% bromophenol blue), and then separated on a 10 or 12% SDS/PAGE gel (standard recipe) and stained with Coomassie blue. For immunoblots, proteins were transferred to a nitrocellulose membrane (Pierce) and blocked with 5% milk in Tris‐buffered saline (TBS). The membrane was incubated with a 1 : 1 combination of OMP26 antisera (collected from mice immunized with OMP26 with aluminum hydroxide adjuvant) and PD antisera (collected from mice immunized with PD with aluminum hydroxide adjuvant), each diluted 1 : 1000 in 1% milk and TBS, followed by incubation with horseradish peroxidase (HRP)‐conjugated goat anti‐mouse IgG (Bethyl Laboratories, Montgomery, TX, USA) at a 1 : 12 000 dilution in 1% milk and TBST (TBS with 0.05% Tween‐20). The membranes were washed with TBS or TBST between antibody incubations. Blots were detected on a Bio‐Rad ChemiDoc Imaging System (Bio‐Rad Laboratories, Hercules, CA, USA) using the LumiGLO Reserve HRP chemiluminescent substrate kit (KPL, Gaithersburg, MD, USA) according to the manufacturer's instructions.

### Protein formulations

Protein antigens were expressed in *E. coli* and purified, as described above. For multi‐subunit vaccines, protein antigens were combined into a single formulation and mixed well, and aliquots were stored at −80 °C. For each vaccine dose, an aliquot was thawed on ice before use. Aluminum hydroxide was added to each formulation just prior to vaccination and gently mixed. All vaccine formulations were kept on ice until ready for injection. To determine changes to antigen solubility in the multi‐subunit formulation, we prepared three solutions, all with aluminum hydroxide adjuvant and at the same concentrations as in the vaccine formulations: OMP26 and PD, PD alone, and OMP26 alone. We separately prepared 4 m ammonium phosphate in 10 mm Tris pH 7.0 buffer and then titrated the ammonium phosphate into each of the three samples in 5 μL aliquots (15‐min incubations).

### Vaccination of mice

Six‐ to eight‐week‐old C57BL/6J mice (both sexes) were vaccinated intramuscularly (IM) with 50 μL (25 μL per hind leg) of recombinant purified PD (10 μg per vaccine dose), OMP26 (10 μg per vaccine dose), or P6 (10 μg per vaccine dose) as individual antigens. Combinations of the proteins were also used as vaccines at 10 μg of each protein per vaccine dose. All vaccine formulations were prepared with the addition of 25 μg aluminum hydroxide (alum) per vaccine dose as adjuvant. Control mice were vaccinated with 25 μg aluminum hydroxide per dose. In some experiments, mice were injected by the intraperitoneal (i.p.) route, and in other experiments, mice were vaccinated with different doses of proteins, 1 or 5 μg instead of 10 μg.

Mice were given the first vaccine dose and then a second dose 7 days later, followed by a third vaccination 14 days after the second dose. Blood was collected from the mice 2 weeks after the second dose of vaccine and/or 2 weeks after the third dose of vaccine, and serum was isolated by centrifuging the blood at 2000 ***g*** for 20 min at room temperature. Serum was used to measure antibody levels to the vaccine antigens.

All mouse experiments were conducted in the Rochester General Hospital Research Institute animal laboratory (registered with and inspected by the USDA) in compliance with regulations and were approved by Institutional Animal Care and Use Committee of Rochester Regional Health (IACUC protocol number: 2017‐001).

### Antibody levels

Protein‐specific antibody levels were determined by enzyme‐linked immunosorbent assay (ELISA) using purified recombinant proteins diluted to 1 μg·mL^−1^ in bicarbonate coating buffer (0.05 m Carbonate–Bicarbonate, pH 9.6) added (100 μL per well) to a medium‐binding 96‐well plate (Greiner Bio‐One, Frickenhausen, Germany). Plates were incubated overnight at 4 °C. The wells were washed three times (200 μL per well) with wash buffer (PBS/0.1% Tween 20) and then incubated with blocking buffer (200 μL per well) (3% non‐fat milk in wash buffer) for 1 h at 37 °C. The wells were washed three times (200 μL per well) with wash buffer and then incubated with sera samples (twofold serially diluted in blocking buffer) for 1 h at room temperature. Initial sera samples were diluted 1 : 100 in blocking buffer. After three additional washes, wells were incubated with goat anti‐mouse antibody conjugated to HRP, diluted 1 : 5000 in blocking buffer (100 μL per well) for 1 h at room temperature. After three additional washes, HRP substrate (TMB Peroxidase Substrate from KPL) was incubated in each well (100 μL per well) for approximately 15 min. The reaction was stopped using 100 μL per well of 0.1 m phosphoric acid, and the plates were analyzed at 450 nm using a Spectra Max plate reader (Molecular Devices, San Jose, CA, USA) and the Softmax endpoint dilution protocol. The results are reported as endpoint concentrations (defined as the reciprocal of the highest dilution that gave 2 times the absorbance of background or negative control) calculated on the Softmax software using linear regression.

### Statistical analysis

The statistical tests were performed using prism software (Graph Pad, La Jolla, CA, USA). Differences between groups were analyzed by unpaired parametric *t*‐test for antibody levels after converting the levels to log base 10 scale. All bars represent mean values and errors bars represent the standard error of the mean (SEM) values. For the purpose of statistical analysis, undetectable antibody levels in samples were arbitrarily assigned a value equivalent to one‐half the lower limit of detection. *P* values of *P* ≤ 0.05, *P* ≤ 0.01, *P* ≤ 0.001, *P* ≤ 0.0001 were considered significantly different (*, **, ***, ****, respectively).

### Mass spectrometry experiments

Prior to the native mass spectrometry (MS) experiments, the His‐tagged proteins (both PD and OMP26) were buffer exchanged into 200 mm ammonium acetate, pH 6.8 using size exclusion micro Bio‐spin P‐6 columns (Bio‐Rad). Both proteins were diluted to a final concentration of 5 μm, as estimated using Thermo NanoDrop 2000c with a theoretical mass (calculated using ProtParam ExPasy tool) of 40 018.60 Da and an extinction coefficient of 61 310 m ^−1^·cm^−1^ (PD) or 20 569.18 Da and an extinction coefficient of 4470 m ^−1^·cm^−1^ (OMP26) [32].

Nano‐electrospray ionization (nanoESI) mass spectra of PD alone, OMP26 alone, or a 1 : 1 mixture of PD and OMP26 were acquired on a Waters Synapt G2 ion mobility mass spectrometer. Emitter tips were pulled in house with a Flaming Brown P97 tip puller. Mass spectrometer tune settings were set as followed: source temperature, 20 °C; sampling cone: 20 V; capillary voltage: 0.7–0.8 kV; trap collision energy: 4 V; trap wave velocity: 250 m·s^−1^; trap wave height: 3.5 V; IM wave velocity: 375 m·s^−1^; IM wave height: 25 V; transfer wave velocity: 100 m·s^−1^; transfer wave height 2 V; backing pressure: 5 mBar. The traveling wave ion mobility cell was filled with helium gas. Data were processed and analyzed using MassLynx and Driftscope (Waters Corp, Milford, MA, USA).

### Size exclusion chromatography experiments

For PD alone and OMP26 alone, 750 μL of each protein, prepared as described above, was combined with 750 μL of phosphate‐buffered saline (PBS) and briefly mixed by inversion. For the PD‐OMP26 mixture, 750 μL of each protein was mixed and incubated together at 4 °C for 1 h. All proteins were separated on a GE Life Sciences (GE Healthcare, Chicago, IL, USA) HiLoad 16/600 Superdex 200 pg size exclusion column using the following standard procedure. 1.5 mL of sample was loaded onto the 1 mL injection loop and passed over to the column that was pre‐equilibrated with PBS. PBS was used during the separation at a flow rate of 0.5 mL·min^−1^. Fractions corresponding to peaks were selected for SDS/PAGE analysis.

## Results

### Antibody response in mice to individual vs multi‐component vaccines

**Fig. 1:**
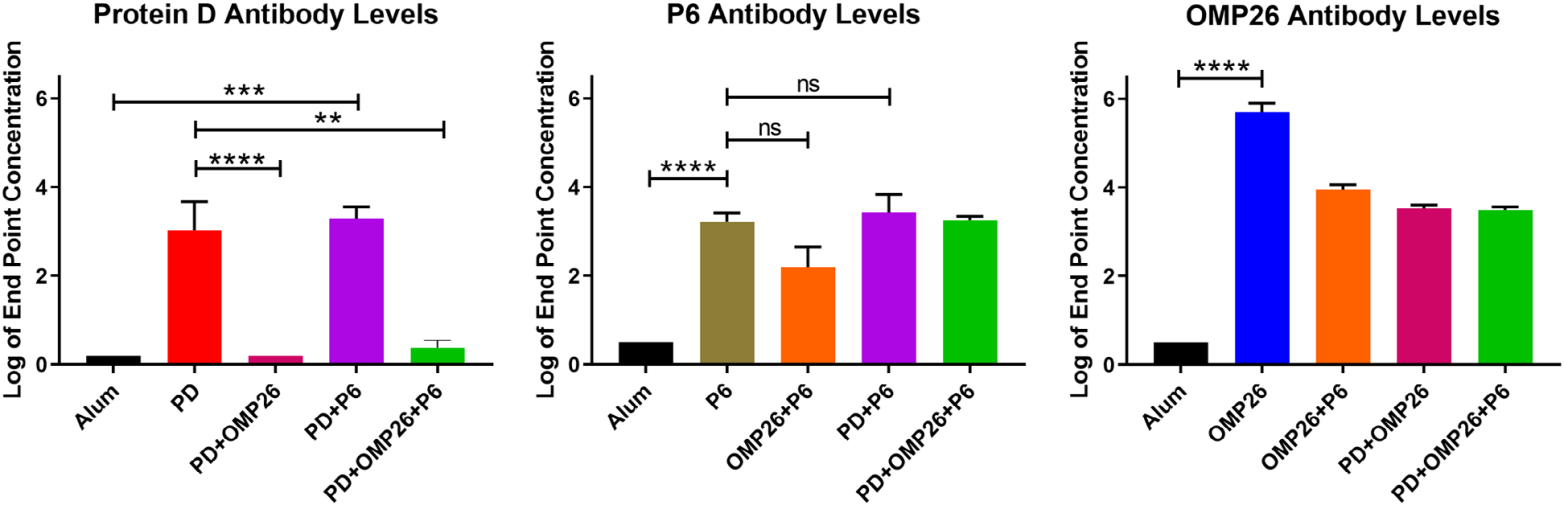
Antibody levels measured with ELISA. All vaccine formulations contain aluminum hydroxide (Alum). Results shown follow three vaccinations to 6‐ to 8‐week‐old C57BL mice with dose 1 at day 0, dose 2 7 days later, and dose 3 14 days after dose 2. Serum was collected 2 weeks after dose 3 and quantitated for antibody to Protein D (PD), P6, or OMP26. Plus sign (+) means the proteins were mixed in equal proportions at 10 μg each. All vaccine groups contained four or five mice, except PD + OMP26 (eight mice). Endpoint levels of antibody in log base 10 are displayed on the vertical axis and vaccine formulations on the horizontal axis. Differences between groups were determined by unpaired parametric *t*‐test. Not all significantly different pairs were shown for better clarity of the figure. Mean and standard error values are displayed. **P ≤ 0.01; ***P ≤ 0.001; ****P ≤ 0.0001, ns, not significant.

Mice immunized with 10 μg PD (four mice) or 10 μg each PD + P6 (five mice) yielded a robust PD antibody response after three doses of antigen (Fig. 1). However, mice immunized with a divalent vaccine mixture formulation containing 10 μg of PD and 10 μg of OMP26 (eight mice) showed little/no antibody response to PD (Fig. 1). Similarly, in a trivalent vaccine formulation containing 10 μg PD, 10 μg OMP26, and 10 μg P6 (five mice), little/no measurable antibody response to PD occurred (Fig. 1). An ELISA confirmed that the presence of OMP26 (1 : 1 mixture) did not decrease the binding of PD to PD polyclonal mouse antibodies in the presence or absence of alum (data not shown). In addition, the presence of OMP26 in the vaccine formulation does not significantly decrease the solubility of PD, as determined by an ammonium phosphate titration (data not shown). Mice immunized with single, divalent, and trivalent vaccines showed measurable antibody responses to P6 and OMP26, as expected (Fig. 1).

### Cobalt affinity purification experiments: Non‐tagged proteins do not co‐purify with His‐tagged proteins

We postulated that OMP26 (homolog to *E. coli* Skp, a chaperone protein) directly or indirectly interfered with PD antibody production by physically binding to PD [33]. To test this hypothesis, we performed cobalt affinity purification experiments.

In preliminary experiments, we found that His‐tagged PD and His‐tagged OMP26 bound to Talon cobalt beads, as expected. Non‐His‐tagged OMP26 did not bind to Talon beads. Non‐His‐tagged PD interacted non‐specifically with Talon beads. Therefore, experiments involving non‐tagged PD were performed in the presence of a low concentration of imidazole (15 mm) to reduce non‐specific binding of non‐tagged PD to the Talon beads.

**Fig. 2:**
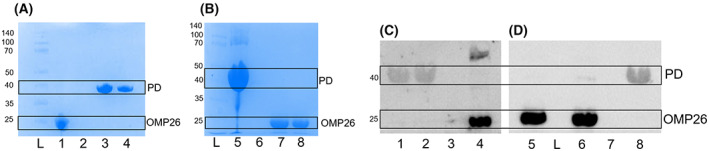
Stained SDS/PAGE gels (A and B) and western blots (C and D) from cobalt affinity purification experiments. Talon beads were incubated with cell lysates for 1 h at room temperature or overnight at 4 °C from *E. coli* cultures of A: (1) His‐tagged OMP26, (2) Non‐His‐tagged OMP26, (3) His‐tagged PD, (4) His‐tagged PD mixed 1 : 1 with non‐His‐tagged OMP26; B: (5) His‐tagged PD, (6) Non‐His‐tagged PD, (7) His‐tagged OMP26, (8) His‐tagged OMP26 mixed 1 : 1 with non‐His‐tagged PD. C: (1) a 1 : 1 mixture of His‐tagged PD and non‐His‐tagged OMP26, (2) His‐tagged PD, (3) non‐His‐tagged OMP26, (4) His‐tagged OMP26; D: (5) a 1 : 1 mixture of His‐tagged OMP26 and non‐His‐tagged PD, (6) His‐tagged OMP26, (7) Non‐His‐tagged PD, or (8) His‐tagged PD. Both blots were developed using a mixture of mouse antisera to PD and OMP26. Molecular weights of ladder markers (L) are in kDa.

When cell lysates from *E. coli* cultures expressing His‐tagged PD and non‐tagged OMP26 were mixed together (1 : 1), non‐tagged OMP26 did not co‐purify with His‐tagged PD (Fig. 2A). When cell lysates from *E. coli* cultures expressing non‐tagged PD and His‐tagged OMP26 were mixed together (1 : 1) in the presence of low levels of imidazole, non‐tagged PD did not co‐purify with His‐tagged OMP26 (Fig. 2B), consistent with failure to detect a physiochemical interaction.

The same experiments were repeated, but samples were run on SDS/PAGE gels and then immunoblotted using PD and OMP26 mouse antisera (collected individually and combined for the western blot). As seen in Fig. 2, non‐His‐tagged OMP26 did not co‐purify with His‐tagged PD (Fig. 2C), and non‐His‐tagged PD did not co‐purify with His‐tagged OMP26 (Fig. 2D), again, consistent with failure to detect a physiochemical interaction.

**Fig. 3:**
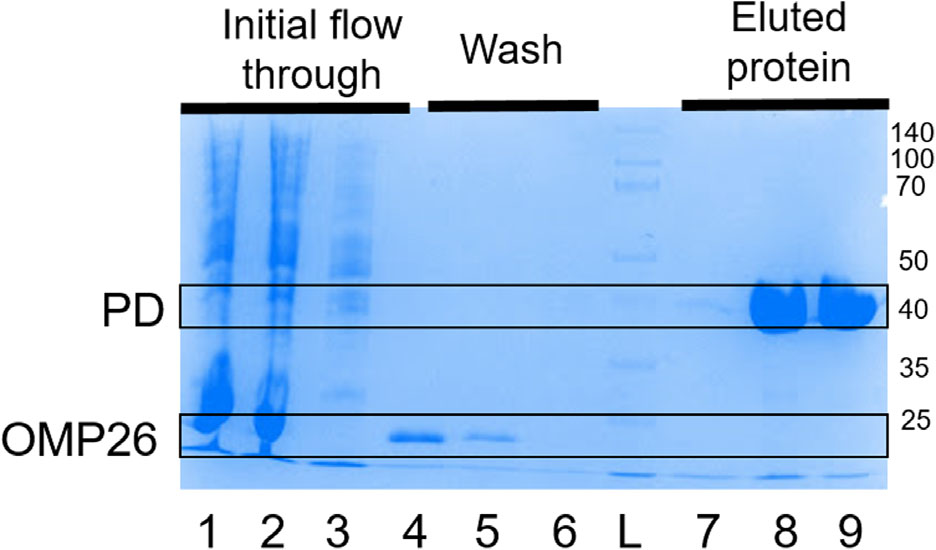
Stained SDS/PAGE gel from a cobalt affinity purification experiment with cell lysates and aluminum hydroxide. Talon beads were incubated with aluminum hydroxide (500 μg·mL^−1^) and a 1 : 1 mixture of cell lysates from *E. coli* cultures overexpressing non‐His‐tagged OMP26 and His‐tagged PD. Lanes 1–6 contain *initial flow‐through* off the columns (1–3) or flow‐through off the column after washing with equilibration buffer (*wash*, 4–6). Fractions eluted from the Talon beads with 150 mm imidazole buffer (*Eluted protein*) were run in lanes 7–9. OMP26 was not present in the eluted fractions following incubation with the 1 : 1 mixture of non‐His‐tagged OMP26 and His‐tagged Protein D. Molecular weights of ladder (L) markers are in kDa.

To test whether or not the adjuvant used in the vaccine formulations was causing the PD‐OMP26 interaction, aluminum hydroxide (500 μg·mL^−1^) was added to the cell lysates of non‐His‐tagged OMP26, His‐tagged PD, or a 1 : 1 mixture of the two. When the individual proteins were run on the Talon column, bands at the approximate molecular weight of OMP26 were seen in the flow‐through and wash fractions from the Talon bead column, while bands at the molecular weight of PD were seen in the elution fractions of the column, as expected. When the 1 : 1 mixture of cell lysates (non‐tagged OMP26 and His‐tagged PD) was run on the Talon column, non‐tagged OMP26 was present in the flow‐through and wash fractions, but not present in the elution fractions, suggesting non‐tagged OMP26 did not co‐purify with His‐tagged PD (Fig. 3), consistent with failure to detect a physiochemical interaction.

The cobalt affinity purification experiments were performed with cell lysates, due to protein isolation challenges with non‐His‐tagged proteins, which was a limitation of this approach. The two additional *in vitro* experiments described below were performed using purified proteins.

### Mass spectrometry experiments: Only homo‐oligomeric states observed in native MS spectra

Native mass spectrometry (MS) preserves three‐dimensional protein conformation, as well as non‐covalent interactions of protein complexes [34, 35, 36]. Gentle ionization conditions were used with minimal collision energy to avoid the fragmentation of potential protein complexes in the gas phase. While native MS informs us of the mass and charge of protein ion species, ion mobility MS separates based on size, shape, and charge of the ions, providing more information about the conformation of the protein. The advantage of using ion mobility in addition to high‐definition MS allows us to distinguish overlapping m/z species of homo‐oligomeric protein complexes such as PD and OMP26. PD alone, OMP26 alone, and a 1 : 1 mixture of PD and OMP26 were sprayed by nano‐electrospray ionization (nESI) to obtain mass spectra. The gentle ionization of nESI produces multiply charged protein species due to protonation, and protein conformations and complexes are considered native‐like.

**Fig. 4:**
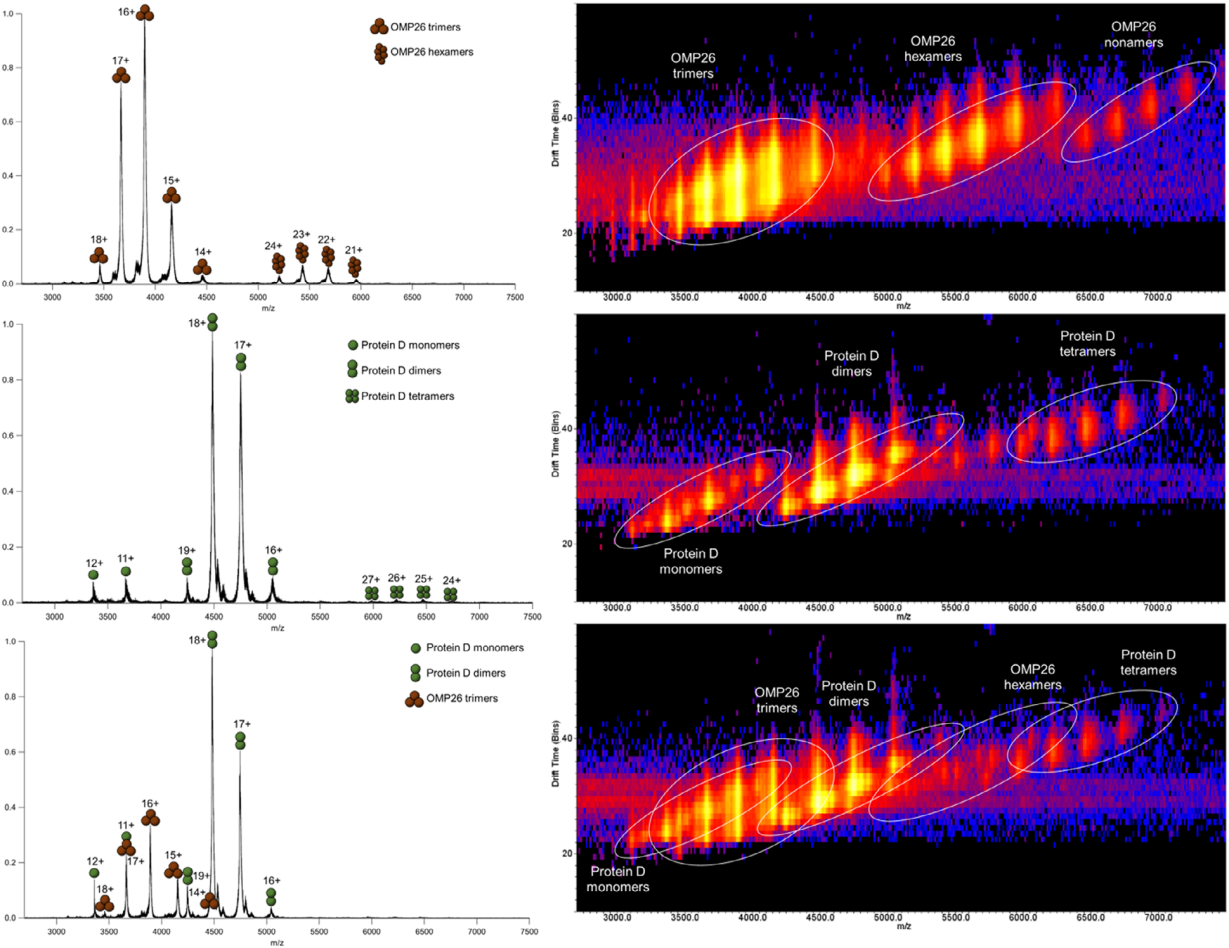
Nano‐electrospray ionization MS and Ion Mobility plots of Protein D and OMP26. (Left, top to bottom) Nano‐electrospray ionization mass spectra of OMP26 alone, Protein D (PD) alone, and a 1 : 1 mixture of PD and OMP26. All proteins were buffer exchanged into 200 mm ammonium acetate, pH 6.8, and diluted to 5 μm in concentration. The predicted charge states and oligomeric states are labeled. (Right, top to bottom) Ion mobility plots of drift time versus *m*/*z* of OMP26 alone, PD alone, and a 1 : 1 mixture of PD and OMP26. The ion species of the predicted protein complexes were circled and labeled accordingly. None of the data were indicative of a PD‐OMP26 complex.

The native mass spectra of PD showed three major oligomeric states of PD: monomers (40,326.1 ± 1.5 Da, charge states +13 to +10), dimers (80,791.4 ± 40.2 Da, charge states +19 to +16), and tetramers (162,205.8 ± 158.5 Da, charge states +27 to +24) (Fig. 4). The native mass spectra of OMP26 indicated that OMP26 forms predominantly trimers (experimental mass of 62 385.1 ± 79.9 Da with charge states of +18 to +14) with low abundance of hexamers (mass_exp_ = 125 213.3 ± 101.6 Da with charge states +24 to +21) (Fig. 4). These experimental masses were in comparison with theoretical masses of OMP26 and PD (20,569.18 Da for OMP26 protomers and 40 841.45 Da for PD protomers). For the 1 : 1 mixture of PD:OMP26, only homo‐oligomeric states of PD and OMP26 individually were observed; no higher‐order species were detected by MS (Fig. 4). Native MS is known to preserve small non‐covalent interactions such as those of salt adducts on the protein complexes, so the current results suggest that the interactions between PD and OMP26 were too weak or insignificant to be captured in the gas phase.

**Fig. 5:**
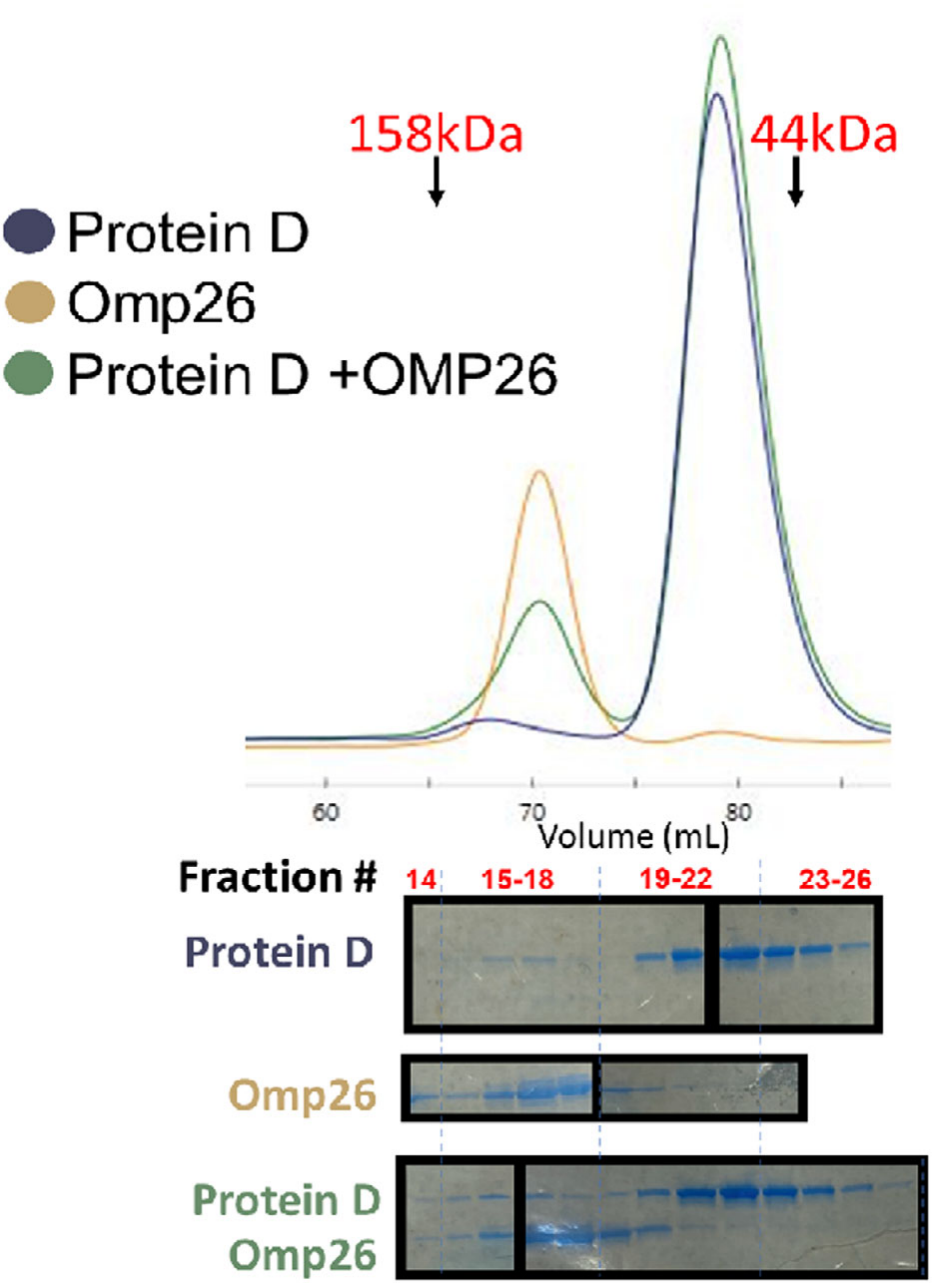
SEC characterization of Protein D (PD), OMP26, and the PD‐OMP26 mixture. Individual PD and OMP26 proteins were purified on Talon beads and then diluted in half with phosphate‐buffered saline (PBS). The PD‐OMP26 1 : 1 mixture was incubated at 4 °C for 1 h. All proteins were separated on a GE Life Sciences HiLoad 16/600 Superdex 200 pg size exclusion column (SEC), pre‐equilibrated with PBS, and then separated at a flow rate of 0.5 mL·min^−1^. SEC chromatogram traces and corresponding SDS/PAGE Coomassie Brilliant Blue stained gels are shown. Gel lanes are positioned under the SEC fractions from which they derive; equal volumes of SEC fractions were loaded into gel lanes, as labeled. Black boxes denote that images were derived from separate gels that were all separated, stained, and destained similarly. Gels were placed on plastic sleeves with white paper inside as a background, and images were captured on a benchtop with an iPhone camera. Approximate peak locations using a molecular size standard are indicated with arrows.

### SEC experiments: Only homo‐oligomeric states observed in SEC

In an alternative approach to determine if the proteins bind to one another to form a hetero‐complex, we performed size exclusion chromatography (SEC) studies in PBS. Fractions from chromatogram peaks were separated by SDS/PAGE, followed by Coomassie blue staining to identify the presence and relative abundance of proteins in those fractions. We observed (Fig. 5): (a) for PD alone, a major peak suggesting a monomer, and a smaller peak suggesting a possible dimer in lower abundance (b) for OMP26 alone, a major peak suggesting a homo‐trimer complex, and (c) for the PD and OMP26 mixture, only peaks similar to those of each individual protein alone and no peak suggestive of alterations in migration and/or the existence of a PD‐OMP26 complex.

### Vaccination experiments: Reversal of PD antibody suppression with separate leg injections

Since the results of our experiments did not support the existence of physiochemical interactions between PD and OMP26 to account for the significantly reduced PD antibody levels, we postulated that antigenic competition might explain the observation. In antigenic competition, the stronger immunogen is taken up and processed at regional lymph nodes more efficiently, resulting in inhibition of expansion of antibody‐producing B cells to the weaker antigen during formation of germinal follicles in the lymph node. Changing the proportion of the ingredients in the vaccine formulation by increasing the concentration of the weaker antigen is a solution that can be pursued.

**Fig. 6:**
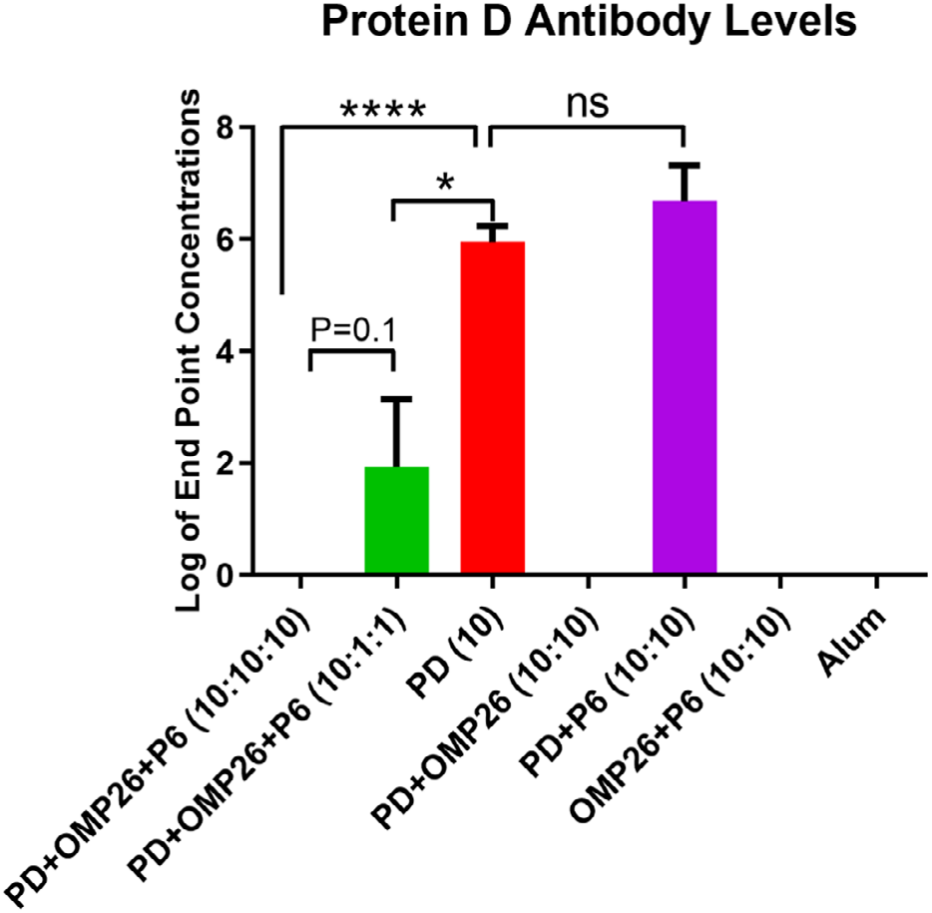
Protein D (PD) antibody levels in response to pre‐mixed vaccines with different PD:Omp26:P6 ratios. All vaccine formulations contain aluminum hydroxide (Alum). The vaccine formulations contain Protein D (PD), OMP26, and/or P6, each at 10 μg per dose or in some formulations 1 μg per dose, as indicated (1 = 1 μg of antigen; 10 = 10 μg of antigen). Results shown follow three vaccinations to 6‐ to 8‐week‐old C57BL mice with dose 1 at day 0, dose 2 7 days later, and dose 3 14 days after dose 2. Serum was collected 2 weeks after dose 3 and quantitated for antibody to PD. All vaccine groups contained five mice, except PD + OMP26 + P6 (10 : 1 : 1) (four mice), OMP26 + P6 (10 : 10) (four mice), PD (10) (three mice), and Alum (four mice). Endpoint levels of antibody in log base 10 are displayed on the vertical axis and vaccine formulations on the horizontal axis. Differences between groups were determined by unpaired parametric *t*‐test. Not all significantly different pairs were shown for better clarity of the figure. Mean and standard error values are displayed. *P* values from the unpaired parametric *t*‐test are labeled. *P ≤ 0.05; ****P ≤ 0.0001; ns, not significant.

We prepared mixed vaccine formulations with different ratios of PD:OMP26, using either 1 or 10 μg of each antigen, and administered two vaccine doses. We found that increasing the ratio of PD:OMP26 from 1 : 1 to 10 : 1 resulted in a 2 log increase in anti‐PD antibody levels (Fig. 6), consistent with antigen competition, although more data points were required to reach significance. PD antibody suppression was not observed when PD was mixed in a 1 : 1 ratio with P6, as shown in prior experiments. The experiments included P6 in the mixtures of PD and OMP26 since our goal was to formulate a trivalent composition of an *NTHi* vaccine (Fig. 6).

**Fig. 7:**
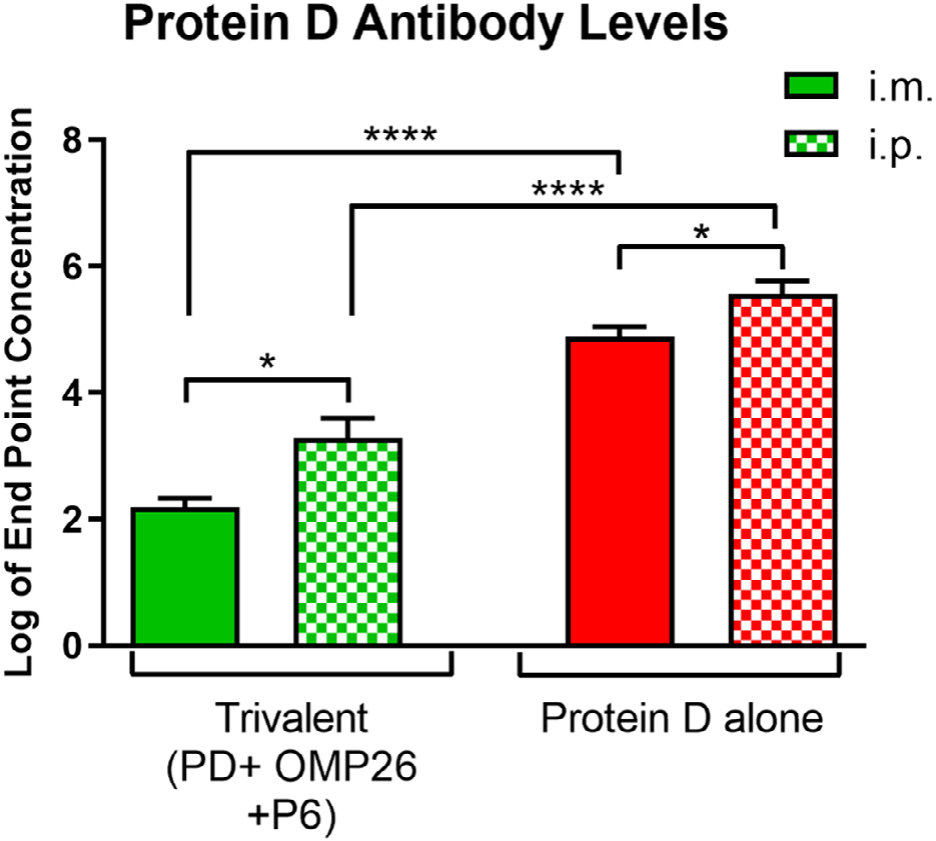
Intramuscular vs. intraperitoneal injections. All vaccine formulations contain aluminum hydroxide (Alum). Vaccine formulations contain Protein D (PD) alone (10 μg per dose) or a mixture of PD, OMP26, and P6, each at 10 μg per dose. Results shown follow three vaccinations to 6‐ to 8‐week‐old C57BL mice with dose 1 at day 0, dose 2 7 days later, and dose 3 14 days after dose 2. Serum was collected 2 weeks after dose 3 and quantitated for antibody to PD. Both trivalent vaccine groups contained five mice, while the i.m. PD group contained six mice and i.p. PD group contained nine mice. Endpoint levels of antibody in log base 10 are displayed on the vertical axis and vaccine formulations on the horizontal axis. Differences between groups were determined by unpaired parametric *t*‐test. Not all significantly different pairs were shown for better clarity of the figure. Mean and standard error values are displayed. *P* values from the unpaired parametric *t*‐test are labeled. *P ≤ 0.05, ****P ≤ 0.0001.

Since antigenic competition is known to occur in regional lymph nodes [37], we conducted an experiment where mice received the proteins by the intraperitoneal (i.p.) route where many lymph nodes would be engaged in antigen processing and production of antibody. Using a trivalent formulation (OMP26:PD:P6 at 10 : 10 : 10 μg per dose) in alum vs. PD in alum alone, we found that i.p. vaccination resulted in 1 log higher anti‐PD antibody levels (*P* ≤ 0.05) compared to i.m. vaccination, although not equivalent to levels for PD alone (Fig. 7), consistent with antigenic competition.

**Fig. 8:**
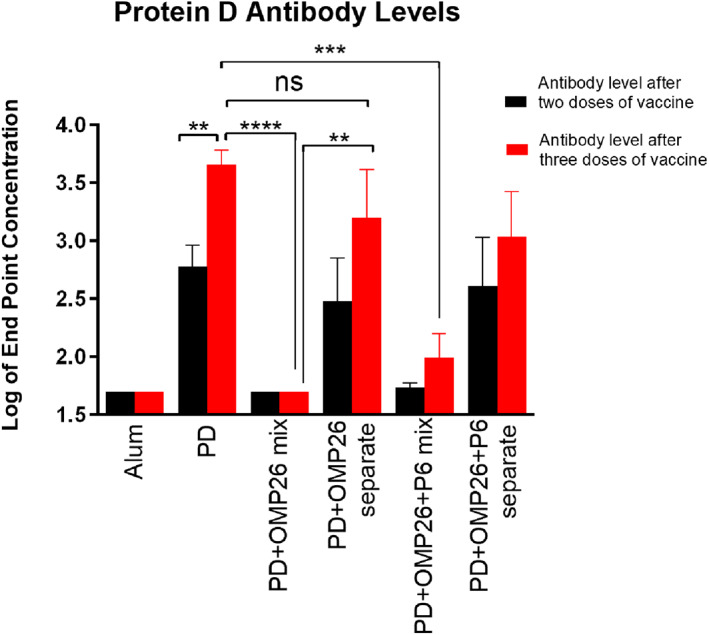
Protein D (PD) antibody levels in response to vaccines given in the same or separate leg injections. All vaccine formulations contain aluminum hydroxide (Alum). Protein vaccines contained one or more of the three antigens (PD, OMP26, P6), each at 5 μg per dose. For PD + OMP26 mix and PD + OMP26 + P6 mix, all listed antigens were mixed into a single formulation and injected into the same leg. For PD + OMP26 separate, PD and OMP26 were injected into separate legs. For PD + OMP26 + P6 separate, PD was mixed with P6 and injected into one leg and OMP26 was injected into the other leg. All vaccines were given to 6‐ to 8‐week‐old C57BL mice with dose 1 at day 0, dose 2 7 days later, and dose 3 14 days after dose 2. Serum was collected 2 weeks after dose 2, prior to dose 3 injections (black), and 2 weeks after dose 3 (red), and quantitated for antibody to PD. All groups contained five mice except PD + OMP26 separate after three doses (four mice). Endpoint levels of antibody in log base 10 are displayed on the vertical axis and vaccine formulations on the horizontal axis. Differences between groups were determined by unpaired parametric *t*‐test. Not all significantly different pairs were shown for better clarity of the figure. Mean and standard error values are displayed. *P* values from the unpaired parametric *t*‐test are labeled. **P ≤ 0.01; ***P ≤ 0.001; ****P ≤ 0.0001; ns (not significant).

In a third mouse vaccination experiment, we delivered the protein antigens separately or in mixtures to regional lymph nodes. Mice were injected with (a) OMP26 in one leg of the mouse and P6 mixed with PD (a mixture that does not cause reduction in anti‐PD antibody) in the other leg or (b) a PD:OMP26 mixture (a mixture that does cause reduction in anti‐PD antibody). We found that mice immunized via separate leg injections of PD and OMP26 yielded similar PD antibody levels to injections with PD alone, whereas the mixture of OMP26 with PD into the same leg, and thereby delivered to the same regional lymph nodes, resulted in significantly reduced anti‐PD antibody levels (Fig. 8), consistent with antigenic competition. OMP26 levels were similar when the vaccines were mixed or administered in separate legs (data not shown).

## Discussion

In this work, we identified a significant reduction in antibody response in mice to PD, a leading vaccine candidate from *NTHi*, when mixed in a formulation that included OMP26, but not P6. OMP26 is homologous to the *E. coli* Skp protein, a chaperone that binds outer membrane proteins in its tentacle‐like hydrophobic helix domain, allowing for efficient transport of OMPs across the periplasm in Gram‐negative bacteria [38]. In light of Skp's biological function, we hypothesized that OMP26 might bind to PD, thereby preventing its interaction with immune cells and inhibiting the PD antibody response. Three separate biochemical strategies (a cobalt affinity purification experiment, SEC, and native MS and ion mobility) were used to test the hypothesis that OMP26 and PD interacted *in vitro*. None of those experiments yielded positive results, suggesting that the two proteins do not interact *in vitro* or that the kinetics of the interaction does not allow for observation of the protein–protein complex using our experimental methods. We therefore pursued an alternative hypothesis that PD mixed with OMP26 led to antigenic competition, with PD being the weaker antigen thereby stimulating much weaker antibody immunity when competing with OMP26. Experimental results using different proportions of the antigens, different routes of immunization to engage more lymph nodes in antigen processing, and different extremity sites of administration to engage different regional lymph nodes, provided support for antigenic competition as the mechanism for the observed effects of PD mixtures with OMP26.

The effectiveness of a vaccine relies on many different properties of the vaccine formulation, including the availability of antigenic epitopes when mixtures of components are prepared and comparable antigenicity to avoid antigenic competition resulting in reduced immunogenicity. Some of these properties are easier to predict than others, and direct *in vitro* and *in vivo* experimentation is needed to address key formulation questions, as we have done here.

Antibody suppression can occur when one or more aspects of the formulation reduce the antibody response to a specific epitope. There are likely different biological scenarios that could result in antibody suppression, and the specific mechanisms of antigenic competition are not well understood. As described by Kim *et al*. [39], antigenic competition may occur due to a deficiency of T‐ and B‐cell interactions to one antigen compared to another, the production of an antibody synthesis inhibitor (perhaps by T lymphocytes), or competition for macrophage surface space between antigens or antigen–antibody complexes. Another interesting example of antibody suppression occurs when pre‐existing immunity to the conjugated carrier molecule in conjugate vaccines, such as PHiD‐CV (from a previous vaccination or exposure), suppresses the overall immune response to the attached antigenic epitopes, a phenomenon referred to as carrier‐induced epitope suppression [40]. Antibody suppression can also occur when a multivalent vaccine is used, leading to lower antibody production to one or more of the antigens, especially when the antigens are highly homologous in sequence or structure [41].

We have previously studied antibody levels to PD, OMP26, and P6 induced by natural exposure to *NTHi* during nasopharynx colonization and AOM in young children 6–30 months of age and found PD responses occurred at a later age than P6, and OMP26 responses were minimal in early child life [24]. However, *NTHi* is not a commensal or pathogen in mice; therefore, pre‐existing antibody to the tested antigens would not play a role to explain our results and indeed pre‐vaccination serum had no detectable antibody to any of the three proteins studied.

Our study has limitations. Since the *in vitro* experiments all produced negative results, we cannot conclude definitively from them that no physiochemical interaction occurs between PD and OMP26. To confirm antigenic competition between PD and OMP26, further experiments to demonstrate preferential OMP26 antigen‐presenting cell uptake, processing, and presentation to both B cells and T cells in regional lymph nodes to illustrate immunodominance are needed.

## Conclusions

Protein D combined with OMP26 in multi‐component vaccine formulation results in significantly lower anti‐PD, but no decrease in OMP26 antibody levels in the serum of mice. The lower levels of PD antibody appear to be due to immunologic antigenic competition and not physiochemical interactions between the proteins.

## Conflict of interest

The authors declare no conflict of interest.

## Author contributions

LVM and RK contributed equally to the study: designing the study, analyzing the data, and writing the manuscript. MG and MH led the SEC and MS sections of the study, respectively, collecting and analyzing the data. LVM and KP performed the mouse vaccination experiments. JP, NJ, IP, AK, and NL performed all protein expression and purification and the cobalt affinity purification experiments. SJ performed the SEC experiments. MP advised on the mouse experiments, interpretation of data, and made revisions to the manuscript.

## Data Availability

The data that support the findings of this study are available from the corresponding author [lvmsch@rit.edu] upon reasonable request. Accession Codes: Protein D: Q06282. P6: P10324. OMP26: Q57483.

## References

[1] Casey JR , Adlowitz DG , Pichichero ME . New patterns in the otopathogens causing acute otitis media six to eight years after introduction of pneumococcal conjugate vaccine. Pediatr Infect Dis J. 2010;29:304–9. 19935445 10.1097/INF.0b013e3181c1bc48 PMC3959886

[2] Kaur R , Casey JR , Pichichero ME . Relationship with original pathogen in recurrence of acute otitis media after completion of amoxicillin/clavulanate: bacterial relapse or new pathogen. Pediatr Infect Dis J. 2013;32:1159–62. 23736142 10.1097/INF.0b013e31829e3779 PMC3845822

[3] Barkai G , Leibovitz E , Givon‐Lavi N , Dagan R . Potential contribution by nontypable *Haemophilus influenzae* in protracted and recurrent acute otitis media. Pediatr Infect Dis J. 2009;28:466–71. 19504729 10.1097/inf.0b013e3181950c74

[4] Pichichero ME . Vaccine‐induced immunologic memory and pace of pathogenesis: predicting the need for boosters. Expert Rev Vaccines. 2008;7:1299–303. 18980532 10.1586/14760584.7.9.1299

[5] Leibovitz E , Asher E , Piglansky L , Givon‐Lavi N , Satran R , Raiz S , et al. Is bilateral acute otitis media clinically different than unilateral acute otitis media? Pediatr Infect Dis J. 2007;26:589–92. 17596799 10.1097/INF.0b013e318060cc19

[6] Vallejo JG , McNeil JC , Hultén KG , Sommer LM , Dunn JJ , Kaplan SL . Invasive *Haemophilus influenzae* disease at Texas children's hospital, 2011 to 2018. Pediatr Infect Dis J. 2019;38:900–5. 31107422 10.1097/INF.0000000000002383

[7] Whittaker R , Economopoulou A , Dias JG , Bancroft E , Ramliden M , Celentano LP , et al. Epidemiology of invasive *Haemophilus influenzae* disease, Europe, 2007–2014. Emerg Infect Dis. 2017;23:396–404. 28220749 10.3201/eid2303.161552 PMC5382729

[8] Klein JO . Role of nontypeable *Haemophilus influenzae* in pediatric respiratory tract infections. Pediatr Infect Dis J. 1997;16:S5–8. 9041620 10.1097/00006454-199702001-00002

[9] Van Eldere J , Slack MPE , Ladhani S , Cripps AW . Non‐typeable *Haemophilus influenzae*, an under‐recognised pathogen. Lancet Infect Dis. 2014;14:1281–92. 25012226 10.1016/S1473-3099(14)70734-0

[10] Swords WE . Nontypeable *Haemophilus influenzae* biofilms: role in chronic airway infections. Front Cell Infect Microbiol. 2012;2:97. 22919686 10.3389/fcimb.2012.00097 PMC3417564

[11] Alikhan MM , Lee FE‐H . Understanding nontypeable *Haemophilus influenzae* and chronic obstructive pulmonary disease. Curr Opin Pulm Med. 2014;20:159–64. 24441573 10.1097/MCP.0000000000000023

[12] Seemungal TA , Hurst JR , Wedzicha JA . Exacerbation rate, health status and mortality in COPD – a review of potential interventions. Int J Chron Obstruct Pulmon Dis. 2009;4:203–23. 19554195 10.2147/copd.s3385 PMC2699821

[13] Vanfleteren LEGW , Spruit MA , Wouters EFM , Franssen FME . Management of chronic obstructive pulmonary disease beyond the lungs. Lancet Respir Med. 2016;4:911–24. 27264777 10.1016/S2213-2600(16)00097-7

[14] Deghmane A‐E , Hong E , Chehboub S , Terrade A , Falguières M , Sort M , et al. High diversity of invasive *Haemophilus influenzae* isolates in France and the emergence of resistance to third generation cephalosporins by alteration of ftsI gene. J Infect. 2019;79:7–14. 31100360 10.1016/j.jinf.2019.05.007

[15] Hara N , Wajima T , Seyama S , Tanaka E , Shirai A , Shibata M , et al. Isolation of multidrug‐resistant *Haemophilus influenzae* harbouring multiple exogenous genes from a patient diagnosed with acute sinusitis. J Infect Chemother. 2019;25:385–7. 30482699 10.1016/j.jiac.2018.09.015

[16] Murphy TF . Vaccines for nontypeable *Haemophilus influenzae*: the future is now. Clin Vaccine Immunol. 2015;22:459–66. 25787137 10.1128/CVI.00089-15 PMC4412935

[17] Murphy TF , Faden H , Bakaletz LO , Kyd JM , Forsgren A , Campos J , et al. Nontypeable *Haemophilus influenzae* as a pathogen in children. Pediatr Infect Dis J. 2009;28:43–8. 19057458 10.1097/INF.0b013e318184dba2

[18] Khan MN , Ren D , Kaur R , Basha S , Zagursky R , Pichichero ME . Developing a vaccine to prevent otitis media caused by nontypeable *Haemophilus influenzae* . Expert Rev Vaccines. 2016;15:863–78. 26894630 10.1586/14760584.2016.1156539

[19] Poolman JT , Bakaletz L , Cripps A , Denoel PA , Forsgren A , Kyd J , et al. Developing a nontypeable *Haemophilus influenzae* (NTHi) vaccine. Vaccine. 2000;19(Suppl 1):S108–15. 11163473 10.1016/s0264-410x(00)00288-7

[20] Michel LV , Kaur R , Zavorin M , Pryharski K , Khan MN , LaClair C , et al. Intranasal coinfection model allows for assessment of protein vaccines against nontypeable *Haemophilus influenzae* in mice. J Med Microbiol. 2018;67:1527–32. 30136923 10.1099/jmm.0.000827

[21] Khan MN , Kaur R , Pichichero ME . Bactericidal antibody response against P6, protein D, and OMP26 of nontypeable *Haemophilus influenzae* after acute otitis media in otitis‐prone children. FEMS Immunol Med Microbiol. 2012;65:439–47. 22463053 10.1111/j.1574-695X.2012.00967.x

[22] Kaur R , Pichichero M . Lipidation of *Haemophilus influenzae* antigens P6 and OMP26 improves immunogenicity and protection against nasopharyngeal colonization and ear infection. Infect Immun. 2022;90:e0067821. 35435727 10.1128/iai.00678-21 PMC9119059

[23] Chang A , Kaur R , Michel LV , Casey JR , Pichichero M . Haemophilus influenzae vaccine candidate outer membrane protein P6 is not conserved in all strains. Hum Vaccin. 2011;7:102–5. 21285530 10.4161/hv.7.1.13351 PMC3062244

[24] Pichichero ME , Kaur R , Casey JR , Sabirov A , Khan N , Almudevar A . Antibody response to *Haemophilus influenzae* outer membrane protein D, P6, and OMP26 after nasopharyngeal colonization and acute otitis media in children. Vaccine. 2010;28:7184–92. 20800701 10.1016/j.vaccine.2010.08.063 PMC3959881

[25] Janson H , Hedén LO , Grubb A , Ruan MR , Forsgren A . Protein D, an immunoglobulin D‐binding protein of *Haemophilus influenzae*: cloning, nucleotide sequence, and expression in Escherichia coli. Infect Immun. 1991;59:119–25. 1987023 10.1128/iai.59.1.119-125.1991 PMC257714

[26] Erwin AL , Smith AL . Nontypeable *Haemophilus influenzae*: understanding virulence and commensal behavior. Trends Microbiol. 2007;15:355–62. 17600718 10.1016/j.tim.2007.06.004

[27] Kyd JM , Cripps AW . Potential of a novel protein, OMP26, from nontypeable *Haemophilus influenzae* to enhance pulmonary clearance in a rat model. Infect Immun. 1998;66:2272–8. 9573117 10.1128/iai.66.5.2272-2278.1998 PMC108191

[28] Janson H , Melhus A , Hermansson A , Forsgren A . Protein D, the glycerophosphodiester phosphodiesterase from *Haemophilus influenzae* with affinity for human immunoglobulin D, influences virulence in a rat otitis model. Infect Immun. 1994;62:4848–54. 7927765 10.1128/iai.62.11.4848-4854.1994 PMC303197

[29] Kyd JM , Cripps AW , Novotny LA , Bakaletz LO . Efficacy of the 26‐kilodalton outer membrane protein and two P5 fimbrin‐derived immunogens to induce clearance of nontypeable *Haemophilus influenzae* from the rat middle ear and lungs as well as from the chinchilla middle ear and nasopharynx. Infect Immun. 2003;71:4691–9. 12874350 10.1128/IAI.71.8.4691-4699.2003 PMC165997

[30] Sveinsdóttir H , Björnsdóttir JB , Erlendsdóttir H , Hjálmarsdóttir MÁ , Hrafnkelsson B , Haraldsson Á , et al. The effect of the 10‐valent pneumococcal nontypeable *Haemophilus influenzae* protein D conjugate vaccine on *H. influenzae* in healthy carriers and middle ear infections in Iceland. J Clin Microbiol. 2019;57. 10.1128/JCM.00116-19 PMC6595461 31068412

[31] Beissbarth J , Smith‐Vaughan HC , Harris TM , Binks MJ , Leach AJ . Use of the 10‐valent pneumococcal *Haemophilus influenzae* protein D conjugate vaccine (PHiD‐CV10) in an Australian Indigenous paediatric population does not alter the prevalence of nontypeable Haemophilus influenzae without the protein D gene. Vaccine. 2019;37:4089–93. 31164306 10.1016/j.vaccine.2019.05.079

[32] Gasteiger E , Hoogland C , Gattiker A , Duvaud S , Wilkins MR , Appel RD , et al. Protein identification and analysis tools on the ExPASy server. In: Walker JM , editor. The proteomics protocols handbook. Totowa, NJ: Humana Press; 2005. p. 571–607.

[33] Walton TA , Sandoval CM , Fowler CA , Pardi A , Sousa MC . The cavity‐chaperone Skp protects its substrate from aggregation but allows independent folding of substrate domains. Proc Natl Acad Sci USA. 2009;106:1772–7. 19181847 10.1073/pnas.0809275106 PMC2644113

[34] Mallis CS , Zheng X , Qiu X , McCabe JW , Shirzadeh M , Lyu J , et al. Development of native MS capabilities on an extended mass range q‐TOF MS. Int J Mass Spectrom. 2020;458:116451. 33162786 10.1016/j.ijms.2020.116451 PMC7641504

[35] Barth M , Schmidt C . Native mass spectrometry‐A valuable tool in structural biology. J Mass Spectrom. 2020;55:e4578. 32662584 10.1002/jms.4578

[36] Heck AJR . Native mass spectrometry: a bridge between interactomics and structural biology. Nat Methods. 2008;5:927–33. 18974734 10.1038/nmeth.1265

[37] Brody NI , Siskind GW . Studies on antigenic competition. J Exp Med. 1969;130:821–32. 4186444 10.1084/jem.130.4.821 PMC2138725

[38] Schiffrin B , Calabrese AN , Devine PWA , Harris SA , Ashcroft AE , Brockwell DJ , et al. Skp is a multivalent chaperone of outer‐membrane proteins. Nat Struct Mol Biol. 2016;23:786–93. 27455461 10.1038/nsmb.3266 PMC5018216

[39] Kim YT , Merrifield N , Zarchy T , Brody NI , Siskind GW . Studies on antigenic competition. 3. Effect on antigenic competition on antibody affinity. Immunology. 1974;26:943–55. 4854459 PMC1423252

[40] Schutze MP , Leclerc C , Jolivet M , Audibert F , Chedid L . Carrier‐induced epitopic suppression, a major issue for future synthetic vaccines. J Immunol. 1985;135:2319–22. 2411793

[41] Hunt JD , Jackson DC , Brown LE , Wood PR , Stewart DJ . Antigenic competition in a multivalent foot rot vaccine. Vaccine. 1994;12:457–64. 7912871 10.1016/0264-410x(94)90125-2

